# Antioxidant Activity and Phenolic Content of *Streblus asper* Leaves from Various Drying Methods

**DOI:** 10.3390/antiox2030156

**Published:** 2013-08-30

**Authors:** Nor Mawarti Ibrahim, Ishak Mat, Vuanghao Lim, Ruzita Ahmad

**Affiliations:** Advanced Medical and Dental Institute, Universiti Sains Malaysia, Bandar Putra Bertam, Kepala Batas, Penang 13200, Malaysia; E-Mails: mawaribrahim@ymail.com (N.M.I.); ddramdi@yahoo.com (I.M.); lvghao@amdi.usm.edu.my (V.L)

**Keywords:** *Streblus asper*, oven dried, freeze dried, DPPH, phenolics, flavonoids

## Abstract

Aqueous and ethanol extracts of oven and freeze-dried *Streblus asper* leaves were investigated using DPPH assay. The presence of phenolic compounds and flavonoids in the extracts, which were detected by Folin and colorimetric assays, respectively, may be responsible for the antioxidant activities of *S. asper*. The different drying treatments resulted in significant differences (*p* < 0.05) in the antioxidant properties as well as the phenolic and flavonoid contents of the *S. asper* extracts. Freeze-dried *S. asper* leaf extracts exhibited high DPPH radical scavenging activity ranging from 69.48% ± 0.03% to 89.25% ± 0.01% at concentrations ranging from 0 to 1 mg/mL, significantly higher compared with the oven-dried extracts which were in the range of 68.56% ± 0.01% to 86.68% ± 0.01%. Generally, the 70% ethanol extract of the freeze-dried samples exhibited higher phenolic and flavonoid content than the aqueous extract, with values of 302.85 ± 0.03 mg GAE/g and 22.70 ± 0.02 mg QE/g compared with 226.8 ± 0.03 mg GAE/g and 15.38 ± 0.05 mg QE/g, respectively. This study showed that *S. asper* leaf extracts contain a number of health promoting bioactive compounds, such as phenolic compounds, and are potential sources of natural antioxidants.

## 1. Introduction

Medicinal plants continue to attract increasing attention because of their potential benefits especially in the field of medicine and pharmacology. Medicinal plants have been recognized for their therapeutic benefits for centuries. Recently, people have started to look for high-quality dried herbal products that are closely associated with the quality of common raw herbal materials. Several factors contribute to the quality of herbs, one of which is color. The final color of a dried plant product is a strong factor for marketing. However, information regarding the specific properties of *Streblus asper* leaves as affected by various drying methods is not available. Studies on the physichochemical characteristics of *S. asper* leaves as a potential herbal supplement are still lacking.

*S. asper* Lour is an herbal plant that belongs to the Moraceae family. This plant is found mainly in surrounding villages and open areas in the northern region of Malaysia. *S. asper* has been used in Malay traditional medicine as decoction and pastes for wound infections. Several studies in Thailand reported that *S. asper* plant alcohol extract possess antibacterial and anti-inflammatory activities [1]. *S. asper* extracts have also been reported to possess anticancer activities. In addition, *S. asper* extract has been traditionally used to treat wounds, skin diseases, filariasis, leprosy, toothache, fever, diarrhea, dysentery and is especially effective in the oral cavity [2,3,4] which has been applied in Ayurveda and folk medicines. Various parts of the *S. asper* plant have been used for different purposes. The bark extract is used to relieve fever, dysentery, toothache and gingivitis; the branch is used as a toothbrush for strengthening teeth and gums; and the leaf exhibits insecticidal activity toward mosquito larvae, antibacterial action, and inhibitory effect on oral and dental diseases [2,4,5].

The potential of *S. asper* leaves as a strong antioxidant has been studied using the 1,1-diphenyl-1-picrylhydrazyl (DPPH) radical scavenging method. DPPH antioxidant assay is based on the ability of the stable free radical DPPH, wherein the deep purple color decolorizes into yellow in the presence of antioxidants. This method has been used to evaluate the free radical scavenging ability of various materials [6] by the degree of discoloration of DPPH [7]. DPPH assay was used in this study because it is one of the most effective, reactive, reliable, simple, and reproducible *in vitro* method for evaluating important activities of compounds, as well as plant extracts [8,9]. Free radicals are molecules, usually of oxygen, which have lost an electron and are continually generated during body metabolism. Free radicals contribute to various health problems, such as cancer, diabetes, hypertension, heart disease, and aging [10]. *S. asper* has been used traditionally for the treatment of heart disease and hypertension [4,11]. Most of the effective antioxidants found in the market are synthetic antioxidants, such as butylated hydroxyanisole (BHA) and butylated hydroxytoluence (BHT) [12]. People are now more concerned about healthy lifestyle, and the demand for safe and natural food preservatives is growing. Thus, the search for potential natural antioxidants has gained increasing interest among researchers. Thus far, very little is known about the antioxidant activity and phytochemical contents of the aqueous and ethanol extracts of *S. asper* leaves using different drying methods. Therefore, this study will compare the effects of two different drying treatments on the phytochemical properties of *S. asper* leaves.

## 2. Experimental Section

### 2.1. Chemicals

DPPH, Folin–Ciocalteu’s phenol reagent, BHA, gallic acid, and quercetin were purchased from MF Chemical Sdn Bhd., Malaysia. Ethanol (EtOH), natrium carbonate, sodium nitrite, aluminum chloride, and sodium hydroxide were purchased from the School of Chemical Sciences, Universiti Sains Malaysia (USM). All chemicals used were of analytical grade.

### 2.2. Collection of Plant Materials

Fresh leaves of *S. asper* were collected from Sungai Petani, Kedah, Malaysia. Botanical identification of these species was carried out by plant botanist, Mr. Shanmugan. Voucher specimen: no 11248 was deposited in the herbarium unit of the School of Biological Sciences, Universiti Sains Malaysia (USM). The samples were divided into powdered oven-dried leaves and powdered freeze-dried leaves. The fresh leaves were cut into small pieces, washed under running tap water, and then dried using either oven or freeze dryer.

### 2.3. Drying Methods

Oven and freeze drying were applied in this experiment to compare the effects of each drying process on antioxidant activity and phenolic properties of *S. asper* leaves. For freeze drying, *S. asper* leaves were dried with a freeze dryer at −50 °C under vacuum (1.6 mmHg) with a pressure of 1.1 × 10^−2^ mB for 72 h and then ground with a dry grinder to obtain fine powdered leaves. For oven drying, *S. asper* leaves were dried in an oven (Memmert, Germany) at 40 °C for 72 h and then ground using the same procedure as that in the freeze-dried powdered leaves.

### 2.4. Samples Extractions

The dried powdered leaves were extracted with water, (30% EtOH), (50% EtOH) and (70% EtOH) using a water bath at temperature 40 °C and 60 rpm for 24 h. Each aqueous and ethanol extract was centrifuged at 40 °C and 1500 rpm for 20 min. The aqueous and ethanol extracts were then freeze dried at −50 °C for 3 days and then evaporated at 40 °C until dry in vacuum, respectively. The crude extracts were stored at −20 °C for further analysis.

### 2.5. *In Vitro* Antioxidant Activity

#### Antioxidant Activity Assay

DPPH scavenging activity was determined by an assay modified previously [13]. The inhibition concentration of sample required to scavenge DPPH radical by 50% (IC_50_) was obtained from a graph plot of percentage inhibitions and extract concentration, using BHA as standard.

### 2.6. Total Phenolics Content

The total phenolic content (TPC) of extracts was measured using the previously reported Folin–Ciocalteu method [14], with slight modifications. The mean (±SD) results of triplicate analyses were expressed as mg of gallic acid equivalents per gram of dry extract (mg GAE/g). The calibration equation for gallic acid was *y* = 0.116*x* (*R*^2^ = 0.9924), where *x* is the gallic acid concentration in mg/mL, and y is the absorbance reading at 725 nm.

### 2.7. Total Flavonoids Content

Total flavonoids content of *S. asper* extracts was determined using the calorimetric method, as described by Sakanaka *et al*., 2005 [15]. The mean (±SD) results of triplicate analyses were expressed as mg of quercetin equivalents per gram of dry extract (mg QE/g). The calibration equation for quercetin was *y* = 0.043*x* + 0.024 (*R*^2^ = 0.980), where x is the quercetin concentration in mg/mL, and *y* was the absorbance reading at 510 nm.

### 2.8. Statistical Analysis

Data were subjected to one-way analysis of variance (ANOVA) and the significance of the difference between means was determined by Duncan’s multiple range test (*p* < 0.05) using the Statistical Package for the Social Sciences (SPSS) Statistics Version 17.0. Values were expressed as mean ± S.D.

## 3. Results and Discussion

### 3.1. Radical Scavenging Assays (RSA)

**Figure 1:**
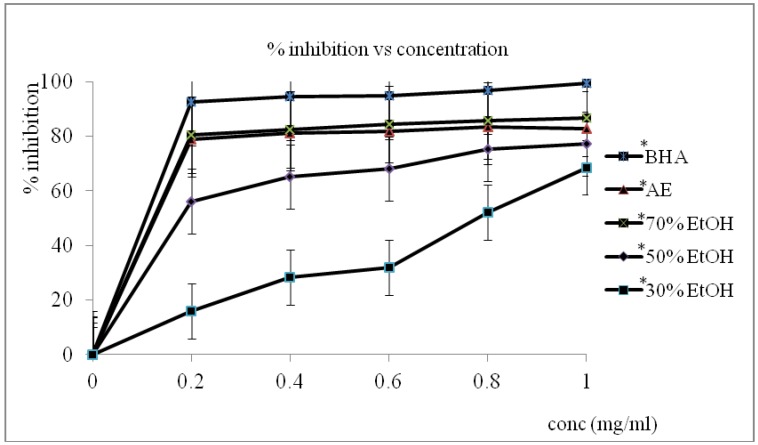
1,1-Diphenyl-1-picrylhydrazyl (DPPH) free radical scavenging activity (%) of the aqueous and ethanol *S. asper* oven dried leaves extract and butylated hydroxyanisole (BHA). Mean ± standard deviation (*n* = 3).

**Figure 2:**
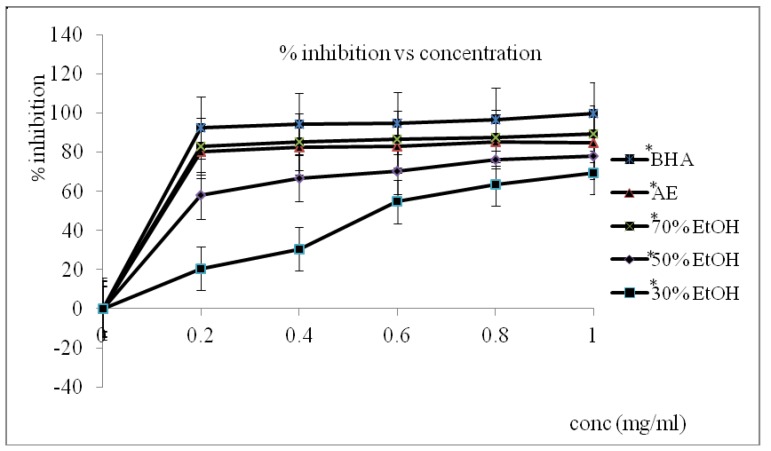
DPPH free radical scavenging activity (%) of the aqueous and ethanol *S. asper* freeze dried leaves extract and BHA. Mean ± standard deviation (*n* = 3).

The antioxidant activities in terms of free radical scavenging activity of the aqueous extract as well as the 30%, 50%, and 70% EtOH extracts of the oven-dried *S. asper* leaves were evaluated using BHA as standard. DPPH free radical assay results expressed as percentages of % inhibition of free radicals in the concentration range of 0 to 1 mg/mL are shown in Figure 1. The scavenging activity of all the samples was highly dependent on concentration, namely, antioxidant activity increased with increase in extract concentration. Results show that the 70% EtOH extract from the oven-dried leaves exhibited the highest scavenging activity with a value of 86.68% ± 0.01% compared with the aqueous extract of *S. asper* leaves, which exhibited 82.88% ± 0.02% scavenging activity. The antioxidant activity was significantly (* *P* < 0.05) different among these extracts in the following order: 70% EtOH > aqueous > 50% EtOH > 30% EtOH. The antioxidant activities of the 50% and 30% EtOH extracts were 77.2% ± 0.01% and 68.56% ± 0.01%, respectively. The extracts from the freeze-dried samples exhibited higher antioxidant activity compared with the oven-dried samples, the 70% EtOH extract of which exhibited higher antioxidant activity (89.25% ± 0.01%), followed by the aqueous extract (84.88% ± 0.01%), the 50% EtOH extract (78.10% ± 0.03%), and the 30% EtOH extract (69.48% ± 0.03%) as shown in Figure 2. The 70% EtOH extract exhibited significantly higher DPPH radical scavenging activity (* *P* < 0.05) compared with the aqueous extract. In this case, 70% EtOH had significant impact compared with the oven-dried extracts. Thus, the highest antioxidant activity among the various solvent extracts was shown by the 70% EtOH extract from the freeze-dried samples (89.25% ± 0.01%), followed by the 70% EtOH extract from the oven-dried samples (86.68% ± 0.01%). BHA had the highest antioxidant activity compared with all extracts for both drying methods. The potential use of *S. asper* leaf extracts as natural antioxidant agent was investigated in this study. Commercial synthetic antioxidants, such as BHT and BHA, exhibit undesirable side effects including toxicity to humans [16]. Thus, new natural bioactive compounds with antioxidant activities that have significant roles for preventing human chronic diseases have been actively researched. This paper is the first report on the antioxidant activity of *S. asper* leaf extracts treated under two different drying methods. The effects of various drying treatments must be evaluated, and the best method of drying must be determined to maintain the antioxidant activity because of the possible high amounts of phenolic compounds such as flavonoids. The extracts of all the tested *S. asper* leaves possessed high radical scavenging activity. Significant differences (* *P* < 0.05) were observed among all the extracts. The freeze-dried aqueous extract exhibited significantly higher radical scavenging activity (* *P* < 0.05) than the oven-dried aqueous extract. In this case, the 70% EtOH extract of the freeze-dried samples exhibited significantly higher activity (* *P* < 0.05) than the oven-dried extracts. Similarly, Lim and Murtijaya [17] found that oven drying led to the significant reduction in the DPPH scavenging ability of *Phyllanthus amarus*. This study also found that the radical scavenging activity of both extracts increased within the concentration range of 0 mg/mL to 1 mg/mL. This result shows that the 70% EtOH leaf extracts are the strongest and potential sources of antioxidant activity, with a percentage of above 80%. This finding is comparable with that of BHA. Both crude extracts exhibited high antioxidant activities, indicating that they are potent antioxidant sources.

* AE = aqueous extract, 70% EtOH = 70% ethanol extract, 50% EtOH = 50% ethanol extract, 30% EtOH = 30% ethanol extract.

* AE = aqueous extract, 70% EtOH = 70% ethanol extract, 50% EtOH = 50% ethanol extract, 30% EtOH = 30% ethanol extract.

### 3.2. Total Phenolic Content

Table 1 shows the TPC of the aqueous, 30% EtOH, 50% EtOH, and 70% EtOH extracts from the different drying methods using the regression equation *y* = 0.116*x* (*R*^2^ = 0.9924), expressed as mg GAE/g of extract. The drying treatments had significant effects (* *P* < 0.05) on the phenolic content of the *S. asper* extracts. Extracts from the oven-dried samples showed that TPC was highest in the 70% EtOH extract (296.37 ± 0.05 mg GAE/g) and lowest in the 30% EtOH extract (146.64 ± 0.03 mg GAE/g). For the aqueous and 50% EtOH extracts, the phenolic contents were 223.95 ± 0.04 mg GAE/g and 155.66 ± 0.02 mg GAE/g, respectively. The TPC from the freeze-dried samples in decreasing order were 302.85 ± 0.03 mg GAE/g (70% EtOH), 226.80 ± 0.03 mg GAE/g (aqueous extract), 163.17 ± 0.01 mg GAE/g (50% EtOH), and 153.09 ± 0.02 mg GAE/g (30% EtOH).

**Table 1:** Total phenolic constituents of *S. asper* leaves aqueous and ethanol extracts from oven dried (OD) and freeze dried (FD) treatments.

Extraction solvent (mL)	Total phenolic content (mgGAE/g)	
	* OD	* FD
AE	223.95 ± 0.04 ^a^	226.80 ± 0.03 ^b^
70% EtOH	296.37 ± 0.05 ^a^	302.85 ± 0.03 ^b^
50% EtOH	155.66 ± 0.02 ^a^	163.17 ± 0.01 ^b^
30% EtOH	146.64 ± 0.03 ^a^	153.09 ± 0.02 ^b^

Data bearing different letters in the same row for each drying method is significantly different (*p* < 0.05); * Mean ± standard deviation (*n* = 3).

We also determined the phenolic contents of the leaf extracts because plant phenolics constitute one of the major compound groups that act as primary antioxidants or free radical terminators. The leaf has been reported to be rich in phenolic compounds [18]. Phenolic compounds are major constituents that have an important role in nutritional values, organoleptic properties, commercial properties, and stabilization of lipid peroxidation due to the scavenging abilities of their hydroxyl group [19]. The result showed that TPC decreased significantly (* *P* < 0.05) during drying. All the extracts of the freeze-dried samples showed significantly (* *P* < 0.05) higher TPC compared with those of the oven-dried samples. TPC of the freeze-dried and oven-dried *S. asper* leaf extracts can be arranged in descending order, as follows: 70% EtOH < aqueous < 50% EtOH < 30% EtOH. Thus, the drying treatments had significant effects (* *P* < 0.05) on the phenolic content of the *S. asper* extracts. According to the literature, freeze drying causes lower phenolic content loss than oven drying [20,21]. In the present study, the antioxidant activities of *S. asper* leaves correlated significantly with DPPH radical scavenging activity and total phenolic compounds (*r*^2^ = 0.929). Good correlation was found between antioxidant activity and phenolic content. This result indicates that the antioxidant activity of *S. asper* leaf extracts may originate from the phenolic compounds. This significant correlation is in agreement with previous findings [22,23,24,25], where a strong relationship was found between antioxidant activity and phenolic content, with phenolic compounds a likely contributor to antioxidant activity. In summary, the results showed that *S. asper* leaves possess a high amount of antioxidants, the majority of which originate from phenolic compounds.

### 3.3. Total Flavonoids Content

The total flavonoid content is reported as quercetin equivalents (QE) in reference to the standard curve: *y* = 0.043*x* + 0.024, *R*^2^ = 0.980. Table 2 shows that drying treatments had a significant (* *P* < 0.05) effect on the flavonoid content. For oven-dried samples, the 70% EtOH extract had the highest phenolic content (22.28 ± 0.03 mg QE/g), followed by the 50% EtOH extract (19.16 ± 0.03 mg QE/g), the aqueous extract (10.26 ± 0.04 mg QE/g), and the 30% EtOH extract (8.87 ± 0.02 mg QE/g). For the freeze-dried samples, total flavonoid contents were found in the order 70% EtOH extract (22.70 ± 0.02 mg QE/g) > 50% EtOH extract (19.74 ± 0.05 mg QE/g) > aqueous extract (15.38 ± 0.05 mg QE/g) > 30% EtOH extract (9.92 ± 0.05 mg QE/g). A comparison of extracts between different drying treatments showed that the 70% EtOH extract of *S. asper* leaves from freeze-dried samples had the highest levels of total flavonoid content. Significant (* *P* < 0.05) effects on the drying treatment of the flavonoid content were also observed. The results showed that the total flavonoid contents from *S. asper* leaf extracts from aqueous, 30% EtOH, 50% EtOH, and 70% EtOH were in the range of 8.87 mg QE/g to 22.70 mg QE/g. Significant differences (* *P* < 0.05) were observed between the oven-dried and freeze-dried *S. asper* leaf extracts. The total flavonoid content in the *S. asper* leaf extracts decreased significantly (* *P* < 0.05) in the oven-dried samples compared with that in the freeze-dried samples. The present result confirms the findings of a previous study [26], which reported that flavonoids might lose their components during heat treatment because of temperature exposure and the duration of the process.

**Table 2:** Total flavonoid constituents of *S. asper* leaves aqueous and ethanol extract from oven dried (OD) and freeze dried (FD) treatments.

Extraction solvent (mL)	Total flavonoid content (mgQE/g)	
	* OD	* FD
AE	10.26 ± 0.04 ^a^	15.38 ± 0.05 ^b^
70% EtOH	22.28 ± 0.03 ^a^	22.70 ± 0.02 ^b^
50% EtOH	19.16 ± 0.03 ^a^	19.74 ± 0.05 ^b^
30% EtOH	8.87 ± 0.02 ^a^	9.92 ± 0.05 ^b^

Data bearing different letters in the same row for each drying method is significantly different (*p* < 0.05); * Mean ± standard deviation (*n* = 3).

Flavonoids and other phenolic compounds are potent water-soluble antioxidants and free-radical scavengers that prevent oxidative cell damage and exhibit strong anti-cancer activity [27,28,29]. Flavonoids have beneficial effects on human health. They are used to treat hypertension and diabetes [30]. Moreover, they possess antioxidant, antimicrobial, anti-carcinogenic [31], and anti-inflammatory activities and effectiveness against diarrhea [32,33]. The presence of flavonoids in the extracts is an indication that *S. asper* has anti-inflammatory [1], anti-diarrheal [34], and antimicrobial [35] activities, and it can also be used for hypertension treatment [4]. Flavonoid intake has a protective role in our diet for the prevention of coronary heart disease [36,37]. Thus, this finding supports the traditional use of *S. asper* extracts in Bangladesh for the treatment of heart disease and cardiac disorders [4,11,38]. However, the correlation of total flavonoid content and antioxidant activity was moderate (*r* = 0.602), whereas low correlation was found between flavonoid content and the total amount of phenolics (*r* = 0.592). This finding indicates that flavonoids had less antioxidant activity than phenolic compounds. Reduction in total phenolic and flavonoid contents due to the different drying treatments were accompanied by a decrease in antioxidant activity. Various drying processes yield different results because of the depletion of naturally occurring antioxidants from raw plant materials [39]. This study showed that freeze-drying is significantly more effective in retaining the phenolics and flavonoids in *S. asper* leaves. Oven drying may degrade phytochemicals and inactivate polyphenol oxidase and other enzymes. In addition, some phenolic compounds may decompose rapidly when dried at elevated temperatures [40]. This finding indicates that aqueous extracts of freeze-dried *S. asper* leaves is a good potential source of natural antioxidants for preventing free radical-mediated oxidative damage, and higher levels of phenolic content are retained in freeze-dried than in oven-dried samples [21]. Reduction in total phenolic and flavonoid contents caused by oven drying was accompanied by a decrease in antioxidant activities because of the long drying period, high temperature, and decline in density and water absorbance capacity [41,42].

## 4. Conclusions

The evaluation of the antioxidant activites and phenolic constituents of *S. asper* leaf extracts showed that the 70% EtOH extracts yield the highest amounts of phenolics, flavonoids and antioxidant potential compared with the other extracts. Processing methods have variable effects on the antioxidant activity and phenolic and flavonoids contents of the *S. asper* leaves. The freeze-dried leaf extracts of *S. asper* showed stronger antioxidant properties and higher amounts of phenolics and flavonoids. These findingsprovide basic data on the potential of *S. asper* extracts for medicinal use. This study is the first report on the antioxidant activities of *S. asper* leaf extracts from different drying treatments. The present findings can be used for further characterization and purification of the crude extracts of *S. asper* leaves.
